# Relationship between nerve fiber layer defect and the presence of epiretinal membrane in a Japanese population: The JPHC-NEXT Eye Study

**DOI:** 10.1038/s41598-019-57260-7

**Published:** 2020-01-21

**Authors:** Atsuro Uchida, Mariko Sasaki, Kaoru Motomura, Kenya Yuki, Toshihide Kurihara, Yohei Tomita, Yoko Ozawa, Kazumasa Yamagishi, Ryo Kawasaki, Akiko Hanyuda, Norie Sawada, Kazuo Tsubota, Shoichiro Tsugane, Hiroyasu Iso

**Affiliations:** 1 Department of Ophthalmology, Keio University School of Medicine, 35 Shinanomachi, Shinjuku-ku, Tokyo 160-8582 Japan; 2 Tachikawa Hospital, 4-2-22 Nishiki-cho, Tachikawa, Tokyo, 190-8531 Japan; 3 National Hospital Organization Tokyo Medical Center, 2-5-1 Higashigaoka, Meguro-ku, Tokyo 152-8902 Japan; 4 Department of Public Health Medicine, Faculty of Medicine, and Health Services Research and Development Center, University of Tsukuba, 1-1-1 Tennodai, Tsukuba, 305-8575 Japan; 5 Ibaraki Western Medical Center, 555 Otsuka, Chikusei, 308-0813 Japan; 6 Department of Vision Informatics (Topcon), Osaka University Graduate School of Medicine, 2-2 Yamadaoka, Suita, Osaka 565-0871 Japan; 7 Epidemiology and Prevention Group, Research Center for Cancer Prevention and Screening, National Cancer Center, 5-1-1 Tsukiji, Chuo-ku, Tokyo 104-0045 Japan; 8 Department of Public Health, Social and Environmental Health, Osaka University Graduate School of Medicine, 2-2 Yamadaoka, Suita, Osaka 565-0871 Japan

**Keywords:** Eye diseases, Epidemiology

## Abstract

The study subjects were residents of Chikusei city, Japan, aged 40 years or older who attended annual health check-up programs and participated in the JPHC-NEXT Eye Study which performed non-mydriatic fundus photography of both eyes. The relationship of glaucomatous fundus changes such as optic disc cupping (cup to disc ratio ≥ 0.7) and retinal nerve fiber layer defect (NFLD) with the presence of epiretinal membrane (ERM) were examined cross-sectionally. A total of 1990 persons gave consent to participate in this study in 2013. The overall prevalence of ERM was 12.9%. Of these, 1755 had fundus photographs of sufficient quality and no history of intraocular surgery (mean age: 62.3 ± 10.0 years). After adjusting for age, sex and refractive error, NFLD was positively associated with the presence of ERM (odds ratio [OR]: 2.48; 95% confidence interval [CI]: 1.24, 4.96; *P* = 0.010), but optic disc cupping was not (OR: 1.33; CI: 0.71, 2.48; *P* = 0.37). The results did not necessarily suggest an association between glaucoma and ERM, but indicated an association between NFLD and ERM.

## Introduction

Epiretinal membrane (ERM) is a semitransparent fibrocellular tissue formed on the surface of the retina, characterized by wrinkling or distortion of the retinal microstructure^1^, and the most common macular diseases and affects millions of people worldwide^2–8^. Clinical manifestations include visual disturbances in the form of metamorphopsia, micropsia, and decreased visual acuity when ERM is localized to the macular or perimacular region^9,10^. Most cases are idiopathic, but others develop secondary to various vitreoretinal conditions such as retinal vascular occlusion, uveitis, trauma, intraocular surgery, and retinal detachment^11,12^.

Glaucoma is a neurodegenerative disease with progressive retinal ganglion cell loss, optic nerve damage, and corresponding visual field loss^13^. It is the second leading cause of blindness globally, and early intervention is essential to reduce irreversible vision loss^14^. Glaucoma can be diagnosed based on thinning of the optic disc rim along with corresponding visual field defects^14^. The presence of retinal nerve fiber layer defect (NFLD) in the superior or inferior arcuate bundles is a useful diagnostic biomarker for glaucoma as NFLD may precede visual field defects^14,15^.

ERM and glaucoma were often found to coexist in the same eye in clinical practice. However, the relationship of these disease is still unclear partly because both ERM and glaucoma are age-related eye diseases and their prevalences gradually increase after age of 40^2–8,16–18^. Several population-based studies have investigated the association between optic disc cupping, one of glaucomatous fundus changes and the presence of ERM, and found no association^7,19,20^. No study has investigated the relationship between NFLD, another glaucomatous change, and the presence of ERM. If this relationship is shown, it would suggest a potential link between the pathologies of glaucoma and ERM and contribute to our understanding of the pathological mechanisms of both diseases. Therefore, we aimed to examine the relationship between glaucomatous fundus changes such as optic disc cupping and NFLD and the presence of ERM to elucidate a potential link under a population-based cross-sectional study.

## Results

### Overall prevalence of ERM

Of the 1990 participants (3974 eyes), 37 were excluded due to missing or suboptimal fundus images of the macula in both eyes. The remaining 1953 participants were used to calculate the overall prevalence of ERM [mean age: 62.4 ± 10.2 years; 797 male (40.8%) and 1156 female (59.2%)].

Any ERM comprising either cellophane macular reflex (CMR) or preretinal macular fibrosis (PMF) was observed in 252 participants, giving a prevalence of 12.9% (Table 1). Based on photographic grading, CMR was present in 8.4% and PMF in 4.6% of participants in this study. The prevalence of ERM continuously increased with age and was 2.6%, 5.3%, 15.3%, 17.9%, and 19.2% in the age groups of 40–49, 50–59, 60–69, 70–79, and >80 years, respectively.

**Table 1:** Age-specific prevalence of epiretinal membrane.

Age range (years)	Number of participants	CMR N (%)	PMF N (%)	Any ERM N (%)
**Men, N (%)**				
40–49	89	0 (0)	1 (1.1)	1 (1.1)
50–59	121	5 (4.1)	0 (0)	5 (4.1)
60–69	327	33 (10.1)	14 (4.3)	47 (14.4)
70–79	223	33 (14.8)	10 (4.5)	43 (19.3)
80+	37	7 (18.9)	1 (2.7)	8 (21.6)
Total	797	78 (9.8)	26 (3.3)	104 (13.1)
**Women, N (%)**				
40–49	140	2 (1.4)	3 (2.1)	5 (3.6)
50–59	218	6 (2.8)	7 (3.2)	13 (6.0)
60–69	482	48 (10.0)	29 (6.0)	77 (16.0)
70–79	275	25 (9.1)	21 (7.6)	46 (16.7)
80+	41	4 (9.8)	3 (7.3)	7 (17.1)
Total	1156	85 (7.4)	63 (5.5)	148 (12.8)
**Total, N (%)**				
40–49	229	2 (0.87)	4 (1.8)	6 (2.6)
50–59	339	11 (3.2)	7 (2.1)	18 (5.3)
60–69	809	81 (10.0)	43 (5.3)	124 (15.3)
70–79	498	58 (11.7)	31 (6.2)	89 (17.9)
80+	78	11 (14.1)	4 (5.1)	15 (19.2)
Total	1953	163 (8.4)	89 (4.6)	252 (12.9)

CMR: cellophane macular reflex, PMF: preretinal macular fibrosis, ERM: epiretinal membrane.

### Characteristics of the participants stratified by the presence of ERM

For the analysis of the association between glaucomatous fundus changes and the presence of ERM, 215 eyes (160 participants) were excluded for missing or suboptimal fundus images of either the optic disc or macula, 97 eyes (72 participants) for concomitant retinal disease, or 405 eyes (233 participants) for a history of intraocular surgery (cataract surgery, retinal surgery including laser photocoagulation and anti-VEGF therapy) (Some cases are overlapped). The remaining 3376 eyes of 1755 subjects [mean age: 62.3 ± 10.0 years; 720 (41.0%) male and 1035 (59.0%) female] were included in this analysis.

Basic characteristics of the study participants stratified by the presence of ERM are shown in Table 2. Participants with ERM were significantly older (P < 0.0001) and had higher diopters in refractive error (*P* = 0.0005). Also, participants with ERM were more likely to have NFLD than those without ERM *(P* = 0.0006). No difference was found in the presence of optic disc cupping (*P* = 0.30), sex (*P* = 0.70), or intraocular pressure (IOP; *P* = 0.78) between participants with and without ERM.

**Table 2:** Basic characteristics stratified by the presence of epiretinal membrane.

	Without ERM	Any ERM	P-value
Participants (N, %)	1633 (93.0)	122 (7.0)	
Age, year	**62.0 (10.1)**	**67.0 (7.1)**	**<.0001**
Sex, Men	672 (41.2)	48 (39.3)	0.70
**Eyes (N, %)**	**3139 (93.0)**	**237 (7.0)**	
Refraction, diopter	−**0.61 (2.53)**	−**0.02 (1.98)**	**0.0005**
IOP, mmHg	14.1 (2.7)	14.0 (2.8)	0.78
Optic disc cupping present	378 (12.0)	34 (14.4)	0.30
NFLD present	**183 (5.8)**	**27 (11.4)**	**0.0006**

ERM: epiretinal membrane, IOP: intraocular pressure, NFLD: nerve fiber layer defect.

Values in table are mean and standard deviation for continuous variables or numbers and percentages for categories. Significant values are in bold.

### Associations between glaucomatous fundus changes and the presence of ERM

After adjusting for age and sex, NFLD was associated with the presence of any ERM (OR: 2.55; 95% CI: 1.28, 5.09; P = 0.008; Model 1 in Table 3). Similarly, after adjusting for age, sex, and refractive error, NFLD was associated with that of any ERM (OR: 2.48; 95% CI: 1.24, 4.96; P = 0.010; Model 2 in Table 3). After excluding eyes with high myopia (< −6 D, 155 eyes), the association remained (OR: 1.81; 95% CI: 1.14, 2.89; P = 0.012). No association was found between optic disc cupping and the presence of any ERM. Similar results were found, stratified by sex (OR: 2.54; 95% CI: 0.95, 6.77; P = 0.063 for men, OR: 2.55; 95% CI: 0.96, 6.73; P = 0.059 for women). Besides, there was the association between NFLD and optic disc cupping after adjusting age, sex and refractive error (OR: 6.73; 95%CI: 4.73, 9.57).

**Table 3:** Multivariate analysis of the relationship between glaucomatous fundus changes and the presence of epiretinal membrane.

	OR	95% CI	p-value
**Optic disc cupping present**			
Model 1	1.32	0.72, 2.42	0.37
Model 2	1.33	0.71, 2.48	0.37
**NFLD present**			
Model 1	**2.55**	**1.28, 5.09**	**0.008**
Model 2	**2.48**	**1.24, 4.96**	**0.010**

NFLD: nerve fiber layer defect.

Model1: adjusted for age and sex.

Model2: adjusted for age, sex and refractive error.

Significant values are in bold.

## Discussion

In this study, we investigated whether glaucomatous fundus changes such as optic disc cupping and NFLD were associated with the presence of any ERM in a Japanese population. We found that NFLD, but not optic disc cupping was associated with the presence of ERM.

Optic disc cupping and NFLD are considered to be major signs of glaucomatous morphological changes. Several population-based studies have investigated the association between optic disc cupping and the presence of ERM, but they did not find any significant associations^7,19,20^. Moreover, there is no comparable population-based study that has investigated the association between the other glaucomatous fundus changes such as NFLD and the presence of ERM.

In this study, we found firstly that NFLD was associated with the presence of ERM, but optic disc cupping was not. Optic disc cupping (cup to disc ratio: CDR) is a valuable marker of glaucomatous change. However, the normal range of CDR values was wide in healthy populations and the interindividual variability of optic disc area was high^21,22^, which made difficult to associate physiological changes or anatomical variations. On the other hand, NFLD indicates the loss of retinal ganglion cells and their axons, and is considered to be a more specific glaucomatous morphological change compared with CDR. In addition, it precedes optic disc cupping^15^. The differences of the features between NFLD and optical disc cupping may lead to this result.

Meanwhile, NFLD can be found in cases other than glaucoma, such as ischemic retinopathies with cotton-wool spots, high myopia^11^, a sequela of optic neuritis due to multiple sclerosis or long-standing papilledema, and others^23^. Moreover, ERM or tangential vitreous traction can distort the retinal nerve fiber layer, which can lead to NFLD. Therefore, the present results merely suggest ERM as one of several possible reasons for NFLD in cases other than glaucoma, although this association was found only after excluding major disorders related to NFLD in cases other than glaucoma, such as retinal disease including ischemic retinopathies, optic disc abnormalities, and high myopia. The results of the present study did not necessarily suggest an association between glaucoma and ERM, but indicated an association between NFLD and ERM.

Although the pathogenesis of neuronal loss in glaucoma has not been elucidated, the activation of microglia may be involved in the initiation of the disease process^24^. Intraocular pressure (IOP) elevation or glaucomatous insult stimulates microglial activation with morphological changes, proliferation, migration, and production of inflammatory cytokines^25^ or reactive oxygen species, ultimately leading to neuronal loss^26,27^. In the retina, inflammation or injury such as tangential traction activates microglia which interacts with Müller cells, followed by gliosis and proliferation^28^, which are suggested to be a main component in ERM formation^29^. Taken together these pathologies might have common process via microglia activation, and cause to form ERM and/or NFLD.

The strengths of this study include the use of standardized grading protocols to define glaucomatous fundus changes and ERM by ophthalmologists including glaucomatous and retinal specialists. The questionnaires enabled detailed analysis of the associations between glaucomatous fundus changes and ERM using information from medical histories. On the other hand, the limitation should be noted. First, we diagnosed ERM according to a standardized grading method using fundus photography but not OCT. To determine the precise prevalence of ERM, both fundus photography and OCT should be used in future studies. Second, we did not evaluate the clinical consequence of glaucomatous fundus changes thoroughly because we did not perform visual field testing.

In conclusion, we analyzed a Japanese cohort with 1990 participants and showed that NFLD was associated with the presence of ERM, but optic disc cupping was not. The results did not necessarily suggest an association between glaucoma and ERM, but indicated an association between NFLD and ERM. Longitudinal studies are warranted to further validate these findings, and if proven, they would contribute to understanding the pathological mechanisms of glaucoma and ERM.

## Population and Methods

### Study population

The JPHC-NEXT Eye Study is an ancillary study of the Japan Public Health Center-Based Prospective Study for the Next Generation (JPHC-NEXT) study^30^. Residents of Chikusei City in Ibaraki Prefecture aged ≥ 40 years who were registered under the national health insurance scheme were invited to participate in annual municipal health check-up programs that included fundus photography. In this analysis, a total of 1,990 individuals who participated in the first year of the baseline survey between May 2013 and September 2013 were included.

Refractive status and IOP were measured using the Tonoref II auto refractometer (Nidek Co., Gamagori, Japan). Non-stereoscopic fundus photographs of both eyes were obtained with the Canon CR-1, a 45° non-mydriatic fundus camera (Canon Inc., Tokyo, Japan) without using pharmacological dilating agents. Images were centered on the optic disc and macula. The presence of NFLD, optic disc cupping, and retinal diseases was determined by two masked ophthalmologists. When they did not agree, the diagnosis was made after further discussion with a third glaucomatous or retinal specialist (K.Y, or M.S.).

First, participants with suboptimal fundus images of the macula in both eyes (dense media opacities or lashes) were excluded from the analysis of the overall prevalence of ERM. Second, for the analysis of the association between glaucomatous fundus changes and the prevalence of ERM, we excluded eyes with suboptimal fundus images of either the optic disc or macula, retinal diseases (age-related macular degeneration, retinal vein occlusion, and diabetic retinopathy), optic disc abnormalities and atrophy, or a history of intraocular surgery (cataract surgery or retinal surgery including laser photocoagulation and anti-VEGF therapy).

The study was conducted in accordance with the Ethical Guidelines for Medical and Health Research Involving Human Subjects, Japan, and approved by the Medical Ethics Committees of the School of Medicine, Keio University, the University of Tsukuba, University of Osaka and the National Cancer Center. Written informed consent was obtained from all individuals who participated in the study.

### Assessment of glaucomatous fundus changes and ERM

The appearance of NFLD on fundus photography was defined as multiple slit-like (wedge-shaped) defects in the superior or inferior arcuate areas located in the temporal half of the retina^15^. Following Gloster and Parry^31^, the vertical C/D ratio was defined as the maximal vertical diameter of the cup to the maximal vertical diameter of the disc (CDR ≥ 0.7)^2^.

The funduscopic appearance of ERM was graded as either early or late stage as described by Mitchell *et al*.^2^. The less severe, early stage was defined by the presence of a thin layer of preretinal cells without visible retinal folds, also termed cellophane macular reflex (CMR). The more severe, late stage was defined by the presence of superficial retinal folds or traction lines with an opaque appearance, also termed preretinal macular fibrosis (PMF). Any membranes outside the macula (a 3000-µm radius centered at the fovea) were graded as negative^2^. We defined a patient as having ERM when CMR or PMF was present in at least one eye.

### Statistical analysis

The prevalence of ERM and baseline characteristics were compiled for all samples and for groups according to the presence of any ERM. Differences in the baseline characteristics between groups were assessed using Student’s t test for continuous variables and chi-squared test for categorical variables.

Associations between glaucomatous fundus changes and the presence of ERM were assessed using two models of multivariable generalized linear mixed model, considering the nested structure of the data such as both eyes of a participant. The first model was to determine the associations adjusting for age and sex. The second model was to determine the associations adjusting for age, sex and refractive error (a substitution for axial length). The results are expressed as odds ratio (OR) with 95% confidence intervals (CI). P-values less than 0.05 were considered statistically significant. All statistical analyses were performed using SAS version 9.4 for Windows (SAS Institute Inc., Cary, NC).
